# Dietary Macronutrient Management to Treat Mitochondrial Dysfunction in Parkinson’s Disease

**DOI:** 10.3390/ijms20081850

**Published:** 2019-04-15

**Authors:** Rijan Bajracharya, Neil A. Youngson, J. William O. Ballard

**Affiliations:** 1 School of Biotechnology and Biomolecular Sciences, University of New South Wales, Sydney, NSW 2052, Australia; rijan.bajracharya@com.au; 2 School of Medical Sciences, University of New South Wales, Sydney, NSW 2052, Australia; n.youngson@unsw.edu.au

**Keywords:** diet, macronutrients, mitochondria, oxidative stress

## Abstract

Mitochondrial dysfunction has been demonstrated to play an important role in the pathogenesis of Parkinson’s disease (PD). The products of several PD-associated genes, including *alpha-synuclein*, *parkin*, *pink1*, *protein deglycase DJ-1*, and *leucine rich repeat kinase 2*, have important roles in mitochondrial biology. Thus, modifying mitochondrial function could be a potential therapeutic strategy for PD. Dietary management can alter mitochondrial function as shifts in dietary macronutrients and their ratios in food can alter mitochondrial energy metabolism, morphology and dynamics. Our studies have established that a low protein to carbohydrate (P:C) ratio can increase lifespan, motor ability and mitochondrial function in a *parkin* mutant *Drosophila* model of PD. In this review, we describe mitochondrial dysfunction in PD patients and models, and dietary macronutrient management strategies to reverse it. We focus on the effects of protein, carbohydrate, fatty acids, and their dietary ratios. In addition, we propose potential mechanisms that can improve mitochondrial function and thus reverse or delay the onset of PD.

## 1. Introduction

Parkinson’s disease (PD) is the second most prevalent neurodegenerative disease in ageing individuals [1]. The classic motor system disturbances such as resting tremor, difficulty initiating movements, and postural instability of the disease [2] are mostly attributed to selective degeneration of dopaminergic (DA) neurons in the substantia nigra pars compacta and the development of neuronal Lewy bodies [3]. The neuronal degeneration is associated with biochemical defects, many of which can be attributed to mitochondrial dysfunction [4]. The role of mitochondria in PD itself came into focus with an observation in the 1980s suggesting that an inhibitor of complex I of the electron transport chain can induce Parkinsonism [5]. Aberrant mitochondrial forms and functions have been identified in a subset of patients with PD [6,7], which demonstrates that mitochondrial dysfunction and oxidative stress have a central role in PD pathogenesis. Mitochondrial dysfunction in the dopaminergic neurons of idiopathic and familial PD is well known although the underlying mechanisms are not clear. Mitochondria are highly dynamic organelles which facilitate many cellular functions such as energy metabolism, the stress response, and cell death. Considering their central role in cellular biochemistry it is not surprising that dysfunctional mitochondria can result in cellular damage and can be linked to a large variety of diseases (e.g., cancer, diabetes, epilepsy) and ageing. In this review, we wish to describe the role of mitochondria in PD pathology and potential treatment via dietary approaches.

Diet is a major contributor to health and disease. A healthy diet can defend the body against certain types of diseases, such as obesity, diabetes, and cardiovascular diseases. Various nutrition guides are published by medical and governmental institutions to educate the public [8,9] on what they should be eating to promote health and even prevent some diseases. However, no such official guidelines have been formed to potentially delay the onset or slow the progression of PD. Accordingly, this is an active area of current research and many studies have examined dietary components that either protect from, or increase the risk of PD. While there have been several reviews on how dietary macronutrients, micronutrients, or supplements can influence PD [10,11,12,13,14,15,16,17,18,19], few of these adequately describe the mechanisms (known or hypothetical) of mitochondrial dysfunction that underlie disease pathology or are reversed by treatment. The goal of this review is to report the current information on how dietary macronutrient management could play a role in maintaining mitochondrial health in PD. We start the discussion by considering animal models of PD because much of the information in this area is from fundamental research that was undertaken in models or *in vitro* systems. To understand the role of dietary management in mitochondrial functions, we then briefly discuss how dietary macronutrients can influence oxidative stress, defects in respiratory complexes, and alter mitochondrial morphology and mitophagy. In this review, we also wish to extend this research area by proposing two potential approaches through which macronutrient composition in the diet could improve PD outcomes via alteration of mitochondria. We note that the interactions between diet and gut microbiota is a developing area of interest [20,21] and these interactions likely influence development of PD [22,23,24,25,26,27]. In the next section, we discuss animal models for PD research, focusing on the *Drosophila* and mouse models. We note other models, including zebrafish (*Danio rerio*) [28,29] as well as non-human primate systems (e.g., reviewed in [30]) have been developed and have provided insight into PD.

## 2. Animal Models for Parkinson’s Disease Research

It is our view that there is no “best model of PD,” as none is a true pathocopy of the human condition. Each of the models is an approximation, each likely holding a certain degree of relevance in a specific circumstance. Thus, translational researchers should: (*i*) understand the strengths and the weaknesses of each model, (*ii*) select the most appropriate model for addressing the experimental question, and (*iii*) apply clinically-relevant designs [31]. 

### 2.1. Drosophila Models for PD

The fruit fly *Drosophila melanogaster* has been crucial for mitochondrial research. The ability to tightly control the reproduction, genetics, and physiology of an animal that shares 75% of its genes with humans has led to many discoveries into normal mitochondrial function as well as its role in disease [32]. Accordingly, *Drosophila* has been an excellent model for studying the role of mitochondria in PD pathology and treatment [33,34,35].

Mutations in *Drosophila* of orthologs of genes that are responsible for human familial PD have recapitulated mitochondrial dysfunction as well as higher order pathologies such as neurodegeneration, motor symptoms and shortened lifespan [36]. Examples of these, gene mutations in the *PINK1*/*Parkin* pathway, *DJ-1*, and *LRRK2* are discussed further below. Additionally, chemically-induced *Drosophila* PD models have been useful, particularly for examination of environmental factors that contribute to disease risk and progression [37]. Further, a combination of these drugs, such as rotenone, MPTP and paraquat, with flies that harbour the PD-associated gene mutations has allowed powerful examination of gene-environment interactions in the disease [38,39,40,41]. Finally, *Drosophila* models have been used effectively in PD-therapy research in drug screens, identification of genetic modifiers that suppress PD, or testing of lifestyle modifications such as diet for management of the disease [41,42,43].

Of course, weaknesses exist in *Drosophila* PD models. Differences in brain anatomy, cardiovascular and respiratory systems, as well as in behaviour and complex motor functions exist. Also, drug metabolism differences restrict the potential for some areas of pharmacological research. In this review, we are focussing on diet–mitochondrial–PD mechanisms which are well conserved between *Drosophila* and humans, particularly in macronutrient utilisation for energy production and potential for generation of oxidative stress.

### 2.2. Mouse Models for PD

Mice are more closely related to humans than *Drosophila*, and so it may be expected that they would be a better model for human health and disease. In terms of PD, the most important signs to replicate are motor symptoms, pathologic accumulation and aggregation of α-synuclein, neuronal cell loss in the basal ganglia, age-related disease progression, and nonmotor symptoms. Unfortunately, none of the current models satisfies all of these criteria. Fortunately, many models fulfil a subset. Empirical choices of adequate models to answer specific questions in translational research need to consider, case by case, the model-specific advantages and limitations [44,45,46].

Recent advances in understanding the pathobiology of PD have been obtained in mice. Kam et al. [47] used an experimentally powerful animal model of Parkinson’s disease and related α-synucleinopathies in which α-synuclein fibrils that are formed *in vitro* (preformed fibrils) are injected directly into the mouse brain. The authors identified a feed-forward loop in which α-synuclein preformed fibrils increased nitric oxide-mediated DNA damage, which in turn activated PAR polymerase 1 (PARP1), generating poly(adenosine 5′-diphosphate-ribose) (PAR) causing cell death through the parthenatos cell-death pathway.

Mouse models have also been instrumental in determining the potential beneficial effects of specific dietary interventions in PD. Perez-Pardo et al. [48] employed a mouse model of PD that injected rotenone or vehicle into the striatum of mice and then started a dietary intervention when motor symptoms were observed. The authors found the rotenone-induced motor and non-motor problems were alleviated by the active diet (AD), which contained precursors and cofactors required for membrane phospholipid synthesis, as well as prebiotic fibres. Most of the nutrients in the AD are part of the nutritional combination known as Fortasyn Connect [49]. Subsequently, the same group showed the combination of the AD and Levodopa showed an additive beneficial effect on rotarod performance [50]. In the next section, we consider oxidative stress in PD. Oxidative stress is the imbalance between the levels of reactive oxygen species (ROS) produced and the ability of a biological system to detoxify these reactive intermediates. Oxidative stress is a key link between PD and diet as most ROS in the cell are generated through mitochondrial metabolism of dietary macronutrients.

## 3. Oxidative Stress in Parkinson’s Disease

Elevated intracellular ROS levels can be toxic and can damage lipids, proteins, and DNA. But low levels of ROS can function as a signalling molecule that activates pathways necessary for normal cell homeostasis [51,52]. Numerous studies have indicated that mitochondrial damage induced by oxidative stress contributes to a series of events leading to degeneration of dopaminergic neurons in PD [53,54]. Also, in both sporadic and genetic causes of PD, oxidative stress is thought to be a common underlying mechanism.

Mitochondria are the primary site of ROS generation within a cell, responsible for more than 90% of the total [24]. Mitochondrial ROS production occurs when electrons leak out of the electron transport chain and react with dioxygen and form superoxide. In response, superoxide dismutase (SOD) in the mitochondria and cytosol converts superoxide to diatomic oxygen and hydrogen peroxide (H_2_O_2_) and is released in the cytosol. The cytosol has peroxisomes containing the enzyme catalase which converts H_2_O_2_ to water thus preventing accumulation of ROS [55]. Mitochondria also contribute to H_2_O_2_ disposal by glutathione peroxidase and glutathione reductase [56,57]. However, an increase in ROS production beyond a threshold, or a failure of the anti-ROS processes contribute to oxidative stress causing cellular and mitochondrial damage. Damage to mitochondria themselves exacerbates the process and can cause a dramatic increase in ROS which can overwhelm the cellular antioxidant mechanisms [58,59]. It has been proposed that enhancing antioxidant function may be useful in treating PD [60]. When used at the appropriate levels the mineral selenium is an important antioxidant and may be involved in combating oxidative stress [61]. Studies found reduced selenium levels in PD patients [62] and selenium reduces bradykinesia, a well-described symptom of PD, in rats [63] suggesting that higher selenium levels may be beneficial for PD patients. More recently, high-throughput allele-specific expression analyses suggested that selenium is beneficial for PD patients through its influence on allelic expression in *LAMP3* [64]. In contrast the benefit of coenzyme Q_10_ is less clear. Two meta-analyses of randomized, placebo-controlled trials found no evidence that coenzyme Q_10_ improved motor-related symptoms or delayed the progression of the disease when compared to placebo [65,66].

The presence of ROS generating enzymes in dopaminergic neurons makes them particularly prone to oxidative stress which may lead to PD. Additionally, dopaminergic neurons contain iron, which can catalyse a Fenton reaction generating superoxide radicals that also can contribute to oxidative stress [67]. This intrinsic sensitivity of dopaminergic neurons to reactive species means that moderate oxidative stress can trigger a series of events that lead to cellular demise. However, importantly, different dietary macronutrients have differing potential for generating oxidative stress, therefore PD could be potentially managed through dietary strategies.

Dietary management has been shown to play a vital role in modulating oxidative stress in various organs. The relative ratio of macronutrients can alter the production of ATP and ROS in mitochondria. Intake of excess protein has been linked to oxidative damage in the pancreas of the mouse by reducing levels of antioxidants such as superoxide dismutase (SOD) [68]. A similar observation of oxidative stress has been reported in rat brains with high protein intake [69]. Conversely, protein restriction reduces mitochondrial ROS levels and DNA and lipid damage in rat livers [70]. These studies suggest the consumption of higher protein causes oxidative damage.

Carbohydrate metabolism will release less ROS than protein and fatty acids for the same amount of ATP produced [71]. However, a high glucose level is generally associated with increased TCA cycle flux and increased ROS levels [72,73], whereas inhibition of mitochondrial pyruvate uptake lowered ROS levels [74,75]. When ROS levels are too high or remain increased during a prolonged period, a vicious circle of ROS-stimulated glucose uptake and glucose-stimulated ROS production can be triggered [71].

The role of fatty acids in the generation of ROS is complicated. One study reported that fatty acid stimulates ROS production through its oxidation [76]. However, ROS generation varies with different types of fatty acids. Medium chain fatty acids were found to induce more mitochondrial ROS generation than long chain fatty acid in rats [77]. The same study also reported differences in saturated or unsaturated fatty acids with unsaturated fatty acids producing more ROS [77]. Moreover, some polyunsaturated fatty acids have anti-inflammatory and neuroprotective properties and have been shown to reduce oxidative stress in various animal models [78,79,80]. Fatty acid availability also alters the lipid packing state of membranes [81] and thus can influence membrane ion permeability. Increased mitochondrial membrane potential is an indication of a less leaky membrane and less ROS production [82,83,84]. Our studies have shown lower levels of basal ROS production in stearic acid (saturated long chain fatty acid) fed diet in *Drosophila* [85]. There are also reports suggesting fatty acid oxidation can drive reverse electron transport and lead to higher mitochondrial generation of ROS [86]. All these studies show different roles of various fatty acids on ROS generation. We propose that the dual function of fatty acids, in meeting energy demand by fatty acid oxidation and through contributing to the composition of cellular and mitochondrial membranes is central to their importance for dietary strategies for management of PD.

## 4. Mitochondrial Respiratory Complex I Deficiency and ROS in Parkinson’s Disease

Studies in the early 1980s provided evidence that substances which inhibit complex I of the mitochondrial respiratory chain (MRC) can cause nigrostriatal degeneration and parkinsonism [87,88,89]. Subsequent studies have found decreased complex I activity in frontal cortex homogenate from individuals with PD [87,90,91]. Further evidence of mitochondrial dysfunction in PD comes from the findings that mutations in genes associated with mitochondrial proteins (*parkin*, *pink1*, and *leucine rich repeat kinase 2 (lrrk2)*) that cause a respiratory complex deficiency, are linked to familial forms of PD [92,93]. Cells derived from patients with *parkin* gene mutations show decreased complex I activity [94]. Mutations in the *pink1* gene also reduce complex I activity [95] and PINK1 is important in remodelling of mitochondrial cristae junctions as well as dopaminergic neuronal survival *in vivo* [96]. Furthermore, α-synuclein, although mostly a cytosolic protein, has been shown to interact with mitochondrial membranes and inhibit complex I in neuronal cultures from a PD patient brain [97]. Changes in subunit proteins and function of mitochondrial complexes II, III, IV, and V have also been reported in PD. However, they are not consistently observed and could be a secondary effect due to some other pathogenic mechanism [94,98,99,100,101]. Therefore, respiratory complex dysfunction has been widely found in PD. Respiratory complex I is the first enzyme of the respiratory chain and contributes to ATP synthesis and maintenance of mitochondrial membrane potential [102,103]. Defects in complex I activity can be expected to produce depressed rates of ATP and cause mitochondrial membrane depolarization leading to excessive generation of ROS [104]. Thus, it would be expected that substantial complex I defects would cause severe, early-onset, rapidly progressive neurological diseases such as PD. Because inhibition of complex I activity is the most consistent finding between human PD and altered mitochondrial respiratory function, dietary modulation of complex I may provide the highest potential for nutritional interventions in PD.

## 5. Relative Macronutrient Content in Diets and Metabolic Flexibility to Reduce Mitochondrial ROS From Complex I

Metabolic flexibility is the capacity of an organism to modify energy oxidation in response to nutrient availability, thus allowing it to adapt to a variety of physiological conditions [105]. Concerning dietary macronutrients, metabolic flexibility enables a cell to favour one substrate (e.g., lipid) over another (e.g., carbohydrate) for energy generation in response to substrate availability, or due to dysfunction in the biochemical pathways of one substrate’s metabolism. The concept of metabolic flexibility has been proposed in the restoration of mitochondrial function in Type 2 Diabetes [106]. A similar approach can be recommended to alleviate complex I defect in PD. Mitochondrial energy (ATP) production from lipid and protein has less reliance on complex I than carbohydrate metabolism [107]. Therefore, strategies that increase fat and protein while reducing carbohydrate metabolism could be considered to be useful for PD that is associated with complex I dysfunction. However, as we discuss later the picture is not that clear as there is evidence that conversely suggests that high carbohydrate diets could be beneficial and high protein diets detrimental for PD. One dietary intervention that involves metabolic flexibility that has been tested in disease with mitochondrial defects is the ketogenic diet i.e., high fat, moderate protein, and low carbohydrate. In balanced diets, the brain generally relies on the metabolism of glucose for energy. However, in the ketogenic diet, ketone bodies produced through fat metabolism in the liver can be used as an alternative fuel for the brain. MPTP (1-methyl-4-phenyl-1,2,3,6-tetrahydropyridine) inhibits complex I activity [5], and a study shows that in MPTP mice model of PD, ketone bodies can exert neuroprotective activity within the brain by promoting antioxidant activity [108]. The ketogenic diet has been shown to increase both glucose metabolism and ATP production in postnatal day 35 male rats [109]. Largely, these improvements have been attributed to ketone bodies being able to act as an “alternative fuel”, ultimately bypassing glycolysis and providing Acetyl-CoA to enter the citric acid cycle and facilitate ATP production [110].

Another potential dietary strategy for management of respiratory complex I defect is to reduce intake of the essential amino acid methionine. Higher intake of methionine has been associated with higher ROS generation in complex I as well as damage to mitochondrial DNA [111]. Amino acids can only generate ATP in a mitochondrial-dependent manner which means that more ROS is produced when proteins are used as the primary fuel for energy. So, methionine restrictive diets or diets with low protein content could be a potential dietary management strategy to reduce complex I defects in PD.

Numerous studies have shown that dysfunction of mitochondria can cause a switch from mitochondrial respiration to aerobic glycolysis, which is also known as a Warburg-like effect [112,113,114]. With regard to PD, a study has shown that parkin deficiency activates glycolysis and reduces mitochondrial respiration [115]. Aw et al., [107] proposed that a diet high in carbohydrate could potentially provide advantages for an organism that harbors a complex I defect if the metabolic substrates of carbohydrates are converted to fatty acids before entry into the mitochondria. In this way, a high carbohydrate diet could meet ATP requirements and also bypass complex I by lipid oxidation. If this is true, it would suggest a potential route for dietary management in diseases with mitochondrial complex I defects such as PD.

## 6. Mitochondrial Dynamics and Morphology in Parkinson’s Disease

Another important mechanistic link between diet and PD is mitochondrial dynamics. Mitochondrial fusion and fission contribute to the maintenance of mitochondrial morphology, function, and help optimize their bioenergetic capacity. Fusion allows the spreading of metabolites, enzymes, and mitochondrial gene products throughout the entire mitochondrial compartment. Mitochondrial fission also plays a major role in the removal of damaged organelles by autophagy [116]. Achieving a balance between fusion and fission allows a cell to have the proper organization of its mitochondrial network during biogenesis and has an important role in muscle adaptation to changing physiological conditions. The ketogenic diet, as well as possessing ketones, also contains medium chain fatty acids and the medium chain fatty acid, decanoic acid, has been associated with an improvement in mitochondrial function/mitochondrial biogenesis as the result of its peroxisome proliferator-activated receptor agonist activity [117]. More recently, a ketogenic diet has also been shown to increase mitochondrial mass and functional competence apparently via peroxisome proliferator-activated receptor γ-coactivator-1α, which regulates mitochondrial biogenesis, mitochondrial sirtuins (such as SIRT3) and the uncoupling protein UCP2 [118].

The main machinery of mitochondrial dynamics consists of three large GTPases that fuse and divide the mitochondrial membranes. They are *mitofusin 1* and *2* (*mfn1* and *mfn2*) (outer membrane fusion), *opa1* (inner membrane fusion) and *dynamin related protein 1* (*drp1*) (fission) [119,120,121,122,123]. Several studies show alteration of mitochondrial morphology in cases of PD. One early study reported a reduction in mitochondrial number and disruption of mitochondrial membrane in muscle tissues of PD patients [124]. There are reports of abnormal mitochondrial distribution, variation in mitochondrial size and swelling in biopsies taken from PD patients [125,126,127,128]. In recent years, PD related genes have been shown to have a critical role in the maintenance of normal mitochondrial morphology with a balance between fusion and fission essential for healthy organelle function. Mutation in *parkin* causes abnormal mitochondrial morphology in fibroblast cells of PD patients [129]. Detailed studies in *Drosophila* have demonstrated that *pink1* and *parkin* act to promote mitochondrial fission or inhibit fusion. And, the phenotypes of *pink1* or *parkin* mutant flies can be dramatically improved by overexpression of *drp1* or down-regulation of *mfn1*/*mfn2* or *opa1* [130,131]. *Parkin* and *pink1* are also involved in the generation of mitochondrial-derived vesicles (MDVs) in response to mitochondrial oxidative stress. *Parkin* binds with MDVs in a *pink1*-dependent manner and targets to lysosomes for degradation in a manner independent of mitophagy [132,133,134]. These findings suggest that *pink1* and *parkin* operate in an early stage to salvage mitochondria by selective extraction of damaged components via vesicular carriers. Similarly, *DJ-1* KO mice accumulate more ROS and have fragmented mitochondria [135]. It has also been shown that overexpressing a mutant form of *α-synuclein* in mice causes abnormalities in mitochondrial structure and function [136].

Fusion and fission machinery plays a vital role in maintaining a healthy mitochondrial population [137]. Mitochondrial dynamics and morphology have a strict interconnection with energy balance and diet [138]. Therefore, the maintenance of mitochondrial morphology could be a key area where dietary management can play an important role. The roles of protein and carbohydrate in mitochondrial morphology have been illustrated by studying morphological changes induced by caloric restriction [139] and starvation [140]. Caloric restriction in mice (40% for 6 months) has been shown to increase mitochondrial fission with no changes in total ATP production [139]. Nutrient starvation of glucose increases fission [140]. However, a very high glucose concentration can also elevate fission through high ROS production [74].

The role of fatty acids and lipid in mitochondrial fusion/fission activity has been widely studied and has revealed differences due to the specific type of fatty acids. A high-fat diet rich in saturated fatty acid (high lard diet) has been reported to facilitate mitochondrial fission by elevating levels of *drp1* and decreasing *mfn2* [141]. However, stearic acid which is also a saturated fatty acid has been reported to promote mitochondrial fusion by stearoylation of transferrin receptor which inhibits JNK signalling leading to reduced ubiquitination of mitofusin [142]. In contrast to the effect of saturated fatty acids (except stearic acid), omega 3 polyunsaturated fatty acids have shown to improve mitochondrial function by reducing ROS production and promoting mitochondrial fusion both with *in vitro* and *in vivo* experiments by increased levels of *mfn2* and ATP levels [143].

## 7. Potential Roles of Macronutrients on the Progression of Parkinson’s Disease

In the above section, we described evidence for how different macronutrients can affect PD, mainly through mitochondrial dysfunction. In this section, we propose that a low protein to carbohydrate (P:C) ratio, and diets rich in certain fatty acids may have high potential for management of mitochondrial dysfunction in PD. We have focussed on these new proposals, and not on previously described diets for management of PD such as the ketogenic diet and caloric restriction as these has been well covered elsewhere [144,145]. Our hypotheses stem from our recent work in *Drosophila parkin* mutants which were initially constructed by Greene et al. [36]. Flies bearing null alleles of *parkin* are viable but exhibit reduced longevity, flight and climbing defects, and male sterility [36]. The locomotor phenotypes result from apoptotic muscle degeneration, while the male sterile phenotype results from a defect in spermatogenesis [36]. One limitation of this model is that it is based on an early onset form of PD known as autosomal recessive juvenile parkinsonism (AR-JP) [146]. AR-JP patients display many of the clinical features of idiopathic PD; however, most cases identified lack Lewy body pathology. This observation has led to the suggestion that Parkin may be required for Lewy body formation, or that dopaminergic neuron loss in idiopathic PD and AR-JP individuals proceed through distinct mechanisms [36].

### 7.1. Protein to Carbohydrate Ratio

**Figure 1:**
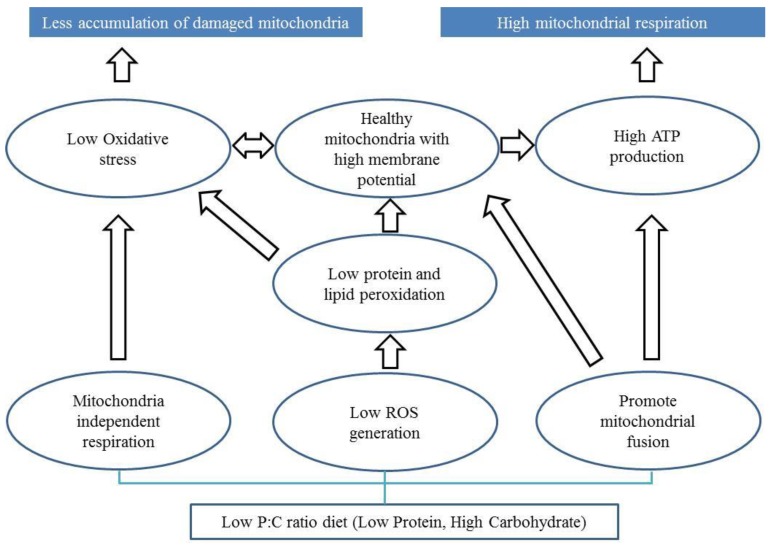
Potential mechanisms by which low protein and high carbohydrate diet can prevent mitochondrial dysfunction in Parkinson’s disease. Low P:C ratio diet can enable mitochondrial independent respiration causing low reactive oxygen species (ROS) generation which may lead to low oxidative stress. Low P:C ratio diet may also facilitate lower ROS generation preventing lipid peroxidation. Low P:C ratio diet can promote mitochondrial fusion and function. The bottom rectangle is the macronutrient (low P:C ratio diet). The ovals represent the potential mechanisms and processes of how macronutrients can affect mitochondrial functions. The top blue filled rectangles are the ways that can lead to healthy and efficient mitochondria in PD.

It has been argued that the ratio or balance of protein to carbohydrate in the food we consume influences ageing [147,148,149,150]. Here we discuss potential mechanisms by which the protein to carbohydrate ratio in the diet may influence the progression of PD. Restricting dietary protein or a balanced diet of carbohydrate and protein has been proposed to improve motor performance and levodopa treatment in PD patients [151,152,153]. However, the role of carbohydrate consumption and PD is still debated. Several lines of evidence suggest the potential for low protein and high carbohydrate diet in improving mitochondrial functions [70,149,154]. However, it is not fully understood if this can improve mitochondrial function in PD patients. We propose three mitochondrial-associated mechanisms that plausibly underlie the benefits of a low P:C ratio diet for PD (Figure 1). 

First, a low P:C ratio diet can facilitate lower ROS levels through a switch in energy production from the mitochondrial-dependent (mitochondrial OXPHOS) to the mitochondrial-independent (glycolysis) pathway. In this way metabolic flexibility therefore avoids the greater potential for carbohydrate metabolism to produce mitochondrial ROS than fat or protein metabolism, and could result in an advantage to patients with a mitochondrial complex I defect [107]. In our recent publication, we showed that a low P:C ratio diet results in low ROS levels and improved mitochondrial functions in a *Drosophila* PD model [42]. However, low ATP generation is a potential limitation of mitochondrial independent respiration.

Second, low ROS generation can lead to low oxidative stress, which can prevent mitochondrial membranes from peroxidation [155,156]. Low lipid peroxidation may prevent the formation of a leaky mitochondrial membrane, which in turn maintains ATP generation [83,157,158].

Third, a low P:C diet may facilitate mitochondrial fusion allowing a greater ATP generation and respiration. Fusion of mitochondria enables the spreading of metabolites, enzymes, and mitochondrial gene products throughout the entire mitochondrial compartment. It can facilitate the extension of mitochondrial health by improving function and ATP generation [116,142,159]. Mitochondrial fusion requires and maintains potential across the inner membrane of mitochondria [160], which can also preserve ATP generation. 

In addition to the potential mechanisms mentioned above, there are studies that show the importance of P:C ratio in determining the composition of gut microbes in dogs and mice [161,162]. Reports have now shown that gut microbes are linked with α-synuclein mediated motor deficits and brain pathology in Parkinson’s disease [163]. Scientists found that the human gut microbes from PD patients can induce motor dysfunction in murid models of PD and concluded that alteration of microbiome can represent a risk factor for PD [162]. The low protein high carbohydrate diet appears to support the growth of *Bacteroides uniformis* and *Clostridium butyricum*, while the high protein low carbohydrate diet increased the abundance of *Clostridium hiranonis*, *Clostridium perfringens*, and *Ruminococcus gnavus* in dogs [161]. But, how these microbes relate to the risk of PD is still unknown, which makes further research into understanding the microbial community a compelling area of future study.

### 7.2. Diets Rich in Saturated Fatty Acids

**Figure 2:**
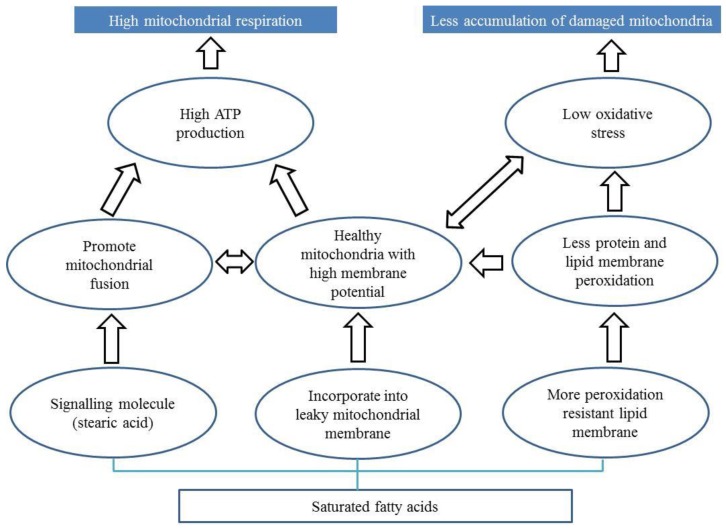
Potential mechanisms by which fatty acid can prevent mitochondrial dysfunction in PD. Fatty acid can be involved in mitochondrial function in three possible ways. First, fatty acid (stearic acid) can act as a signalling molecule to promote mitochondrial fusion. Second, saturated fatty acids can be directly incorporated into leaky mitochondrial membranes. Third, saturated fatty acids can be incorporated into lipid membranes making them resistant to peroxidation. The bottom rectangle is the macronutrient (saturated fatty acid). The ovals represent the potential mechanisms and processes of how the macronutrient can affect mitochondrial functions. The top blue filled rectangles are the ways that can lead to healthy and efficient mitochondria in PD.

The role of fatty acids in the progression of PD is debated in the literature. Here we propose three potential mechanisms by which fatty acids can affect the progression of PD (Figure 2) via alteration of mitochondria.

First, some fatty acids (e.g., stearic acid) may prevent mitochondrial dysfunction in PD by promoting mitochondrial fusion [142] Stearic acid acts a signalling molecule which stearoylates transferrin receptor thereby inhibiting the activation of JNK signalling pathway [142]. This leads to ubiquitination of mitofusin promoting mitochondrial fusion and function. Interestingly, stearoylation of the transferrin receptor has been shown to be specific to stearic acid.

Second, saturated fatty acids may bind to leaky mitochondrial membranes promoting mitochondrial function. Stearic acid is known to be converted to oleic acid [164], which is incorporated into the mitochondrial phospholipid membrane in rats [165]. Plausibly, oleic acid derived from stearic acid may be integrated into these leaky membranes promoting mitochondrial respiration and function. Phospholipids make up the characteristic outer and inner membranes that give mitochondria their shape [166]. Leaky membranes are indicative of lower membrane potential, which in turn can reduce ATP generation [82,83]. Dysfunctional and leaky mitochondria also produce more ROS [84], which can cause an imbalance that can compromise the ability of an organism to detoxify these reactive intermediates causing oxidative stress. So, incorporation of saturated fatty acids likely results in increased membrane potential, improved ATP generation, and reduced oxidative stress.

Third, saturated fatty acid can bind to lipid membranes making them more resistant to peroxidation [81,167]. Membrane peroxidation is observed in *parkin*-deficient mice [168]. Peroxidation resistant membranes are found to be correlated with extended lifespan and mitochondrial function in many organisms such as birds and rats [51,169,170,171,172,173,174]. Supplementation of saturated fatty acid to reduce membrane peroxidation can be a promising approach to mitigate mitochondrial defects in PD patients.

## 8. Conclusions

Improvement of mitochondrial function through dietary approaches is promoted for the management of diseases such as epilepsy, cancer, and diabetes. Considering the growing evidence that mitochondrial dysfunction plays a crucial role in the pathophysiology of PD, it is time to ponder how diets could aid in its management. We suggest that low protein/high carbohydrate diets, or diets enriched with certain fat species could reverse many of the mitochondrial-associated cellular changes in PD. However, as many of the observations that support these dietary approaches stem from animal models or fundamental biochemical research, a cautious approach to human testing is essential. Further, it is not known whether partial nutritional supplementation with specific foods will have therapeutic value. For example, is it possible that regular consumption of a banana or a mango (which have a P:C value of ~1:16) has the potential to delay the progression of PD?
